# Meniscus delivery: a maneuver for easy arthroscopic access to the posterior horn of the medial meniscus

**DOI:** 10.1051/sicotj/2016007

**Published:** 2016-03-16

**Authors:** Hatem Galal Said, Saumitra Goyal, Tarek Nabil Fetih

**Affiliations:** 1 Orthopaedic Department, Assiut University Hospital, Assiut University Assiut Egypt

**Keywords:** Knee arthroscopy, Arthroscopic meniscectomy, Posterior horn, Medial meniscus, Meniscus delivery

## Abstract

Pathology of posterior horn of medial meniscus is common and often presents a difficult approach during arthroscopy for various reasons. We describe an easy maneuver to facilitate “delivery of the medial meniscus” during arthroscopy.

## Introduction

Treatment of medial meniscus pathology is one of the most commonly performed arthroscopic and often presents difficult access during arthroscopy [1, 2]. There are various anatomical constraints in that the medial meniscus is mobile and the medial femoral condyle obscures the view of a large portion of the meniscus which is more convex and larger posteriorly when compared to lateral counterparts. Access to the posterior horn of the medial meniscus often requires a valgus stress while holding the knee slightly flexed and externally rotated to open medial joint [3–5]. This becomes quite difficult in arthritic knees and may result in a requirement for additional portals [6] or tools such as a needle [7] or suture punch [8, 9], and leaving the operating surgeon frustrated. Partial release of the medial collateral ligament (MCL) can improve medial joint opening but iatrogenic valgus instability is a risk [10–12].

We describe a technique to improve medial meniscus posterior horn accessibility without use of additional portals or tools.

## Surgical technique

**Figure 1.**
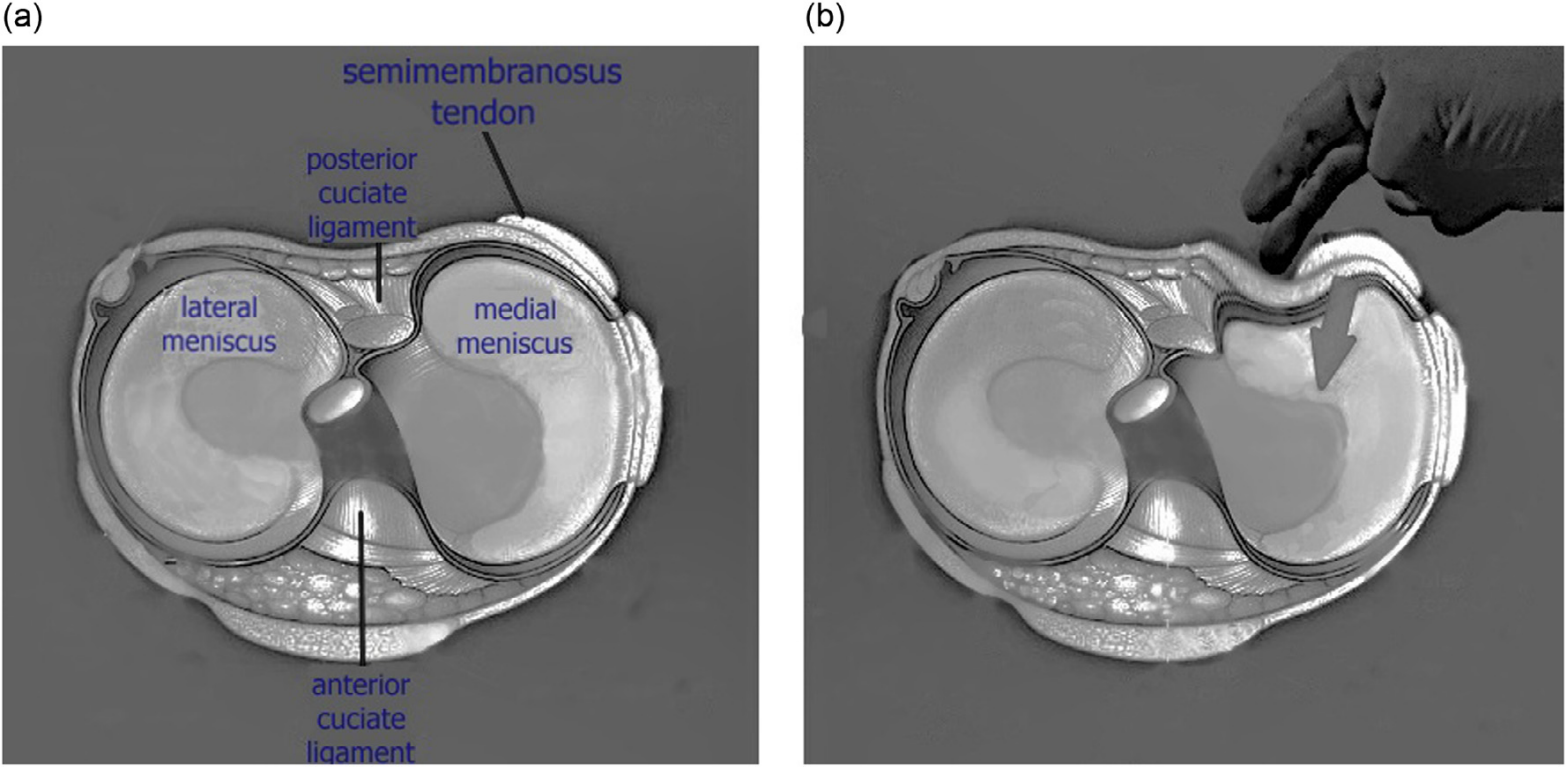
(a, b) The assistant places the middle or index finger on the posterior knee surface just medial to the semimembranosus tendon at the level of the joint line, and applies anteriorly directed pressure delivering the posterior horn of the medial meniscus into view.

**Figure 2.**
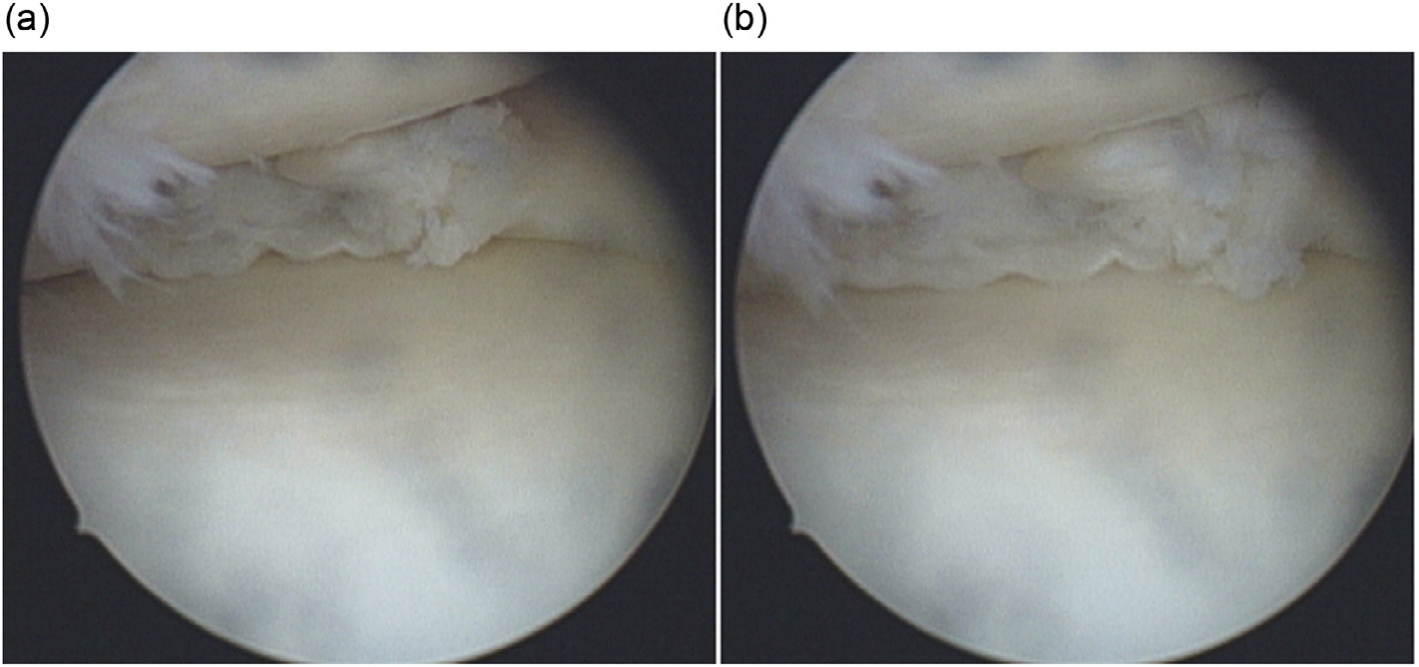
(a, b) Arthroscopic image of left knee showing the position and tear of posterior horn of medial meniscus with and without the maneuver.

**Figure 3.**
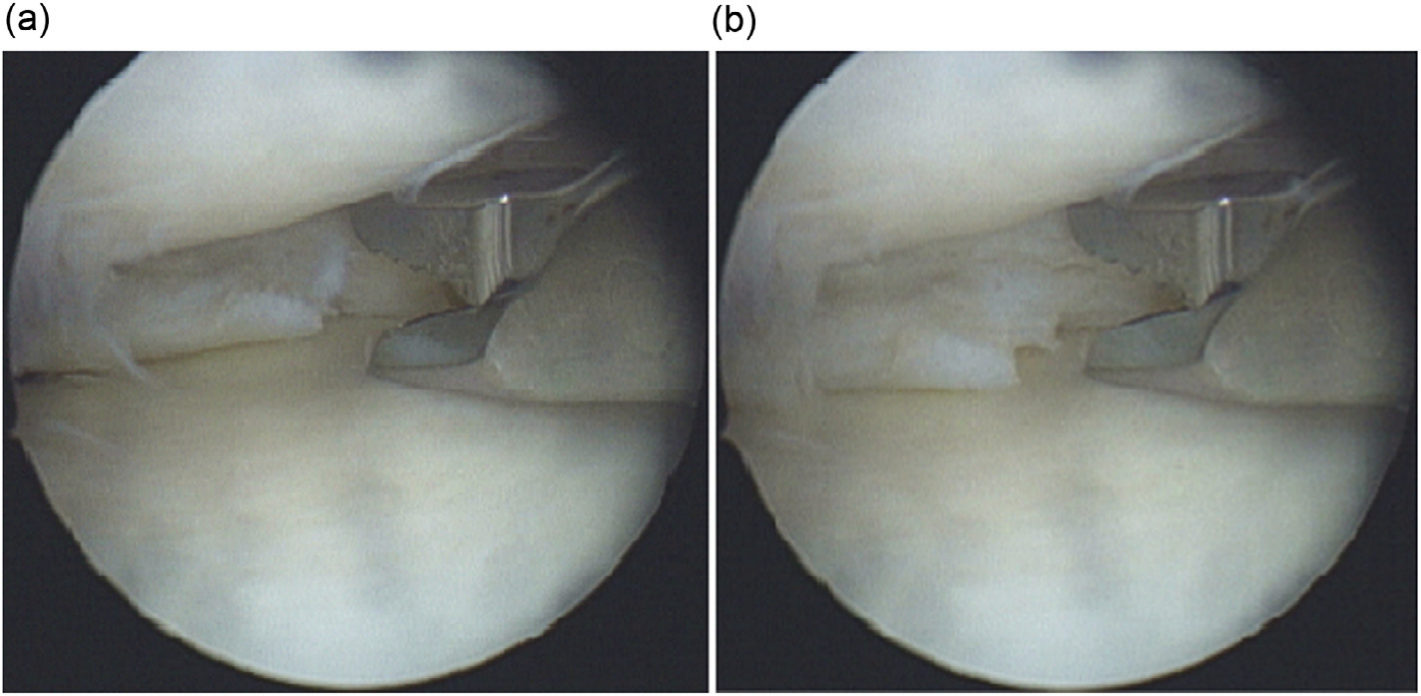
(a, b) Arthroscopic image of left knee showing approach of basket punch to the posterior horn of medial meniscus with and without the maneuver.

Standard anterolateral and anteromedial portals are used to enter the knee joint. Lateral compartment procedures are completed as it can be easily enlarged by conventional techniques. Medial compartment is assessed and configuration of medial meniscus tear is established through the anterolateral portal. The degree of opening of the medial joint is assessed by valgus stress in extension and slight internal rotation of the knee. If the standard 4 mm shaver can enter the posteromedial corner of the knee the space is usually adequate to access the posterior horn. Stable flap tears in the posterior horn can easily be resected using a cutting punch and oscillating shaver. The peripheral portion or unstable flaps become difficult to reach due to “posterior slipping away” of the posterior horn of the meniscus especially in cases of menisco-capsular separation. In this scenario we use the technique of “meniscus delivery”. With the knee in 20–30 degrees of flexion, valgus stress is applied and the medial joint is allowed to open as much as possible, then the assistant places the middle or index finger on the posterior knee surface just medial to the semimembranosus tendon at the level of the joint line, and applies anteriorly directed pressure towards the centre of knee joint (Figure 1). This delivers the posterior horn of the medial meniscus into view which gives a better accessibility and prevents posterior “slipping away” during meniscal resection (Figures 2 and 3).

## Discussion

It is often difficult to visualize the posterior horn of the medial meniscus during arthroscopy and struggling to enter this part of the compartment may lead to iatrogenic cartilage injury and/or instrument damage. The maneuver we describe uses routine tools and allows easy access to the posterior horn without the need for additional portals or special tools. The push to the posteromedial aspect of joint must be done in the valgus opened knee and this allows delivery of the posterior horn into view for easy accessibility. This technique takes no extra time and is especially helpful in tight knees and osteoarthritic knees. It is described for the medial meniscus only as the lateral meniscus is usually easy to approach by conventional methods.

## Conflict of interest

The authors declare no conflict of interest in relation with this paper.

## Supplemental Material


**Video 1**. Meniscus delivery.
**Video 2**. Partial resection using basket and shaver with posterior horn delivered.
